# Suitability of Protein Content Measured by MilkoScan FT-Plus Milk Analyzer to Evaluate Bovine and Ovine Colostrum Quality

**DOI:** 10.3390/ani11092587

**Published:** 2021-09-03

**Authors:** Anna Antonella Spina, Carlotta Ceniti, Francesca Trimboli, Domenico Britti, Vincenzo Lopreiato

**Affiliations:** 1 Interdepartmental Services Centre of Veterinary for Human and Animal Health, Department of Health Science, Magna Græcia University, 88100 Catanzaro, Italy; aa.spina@unicz.it (A.A.S.); ceniti@unicz.it (C.C.); trimboli@unicz.it (F.T.); britti@unicz.it (D.B.); 2 Department of Animal Sciences, Food and Nutrition, Faculty of Agriculture, Food and Environmental Science, Università Cattolica del Sacro Cuore, 29122 Piacenza, Italy

**Keywords:** colostrum, IgG, MilkoScan FT^+^, RID

## Abstract

**Simple Summary:**

A rapid and reliable method for assessing colostrum quality is needed to ensure enough immunoglobulins available to offspring in order to avoid the failure of the passive transfer of immunity. This study evaluates MilkoScan FT-plus as a rapid tool for the evaluation of immunoglobulin content indirectly by protein content measurement and thus colostrum quality with a particular focus on bovine and ovine colostrum. The results indicate that MilkoScan FT-plus has good agreement with the reference method for the measurement of immunoglobulin concentration in colostrum represented by the radial immunodiffusion method.

**Abstract:**

The objective of this study was to evaluate MilkoScan FT-plus for the estimation of the immunoglobulin G (IgG) content in bovine and ovine colostrum. Between April and May 2016, a total of 94 colostrum samples (54 from Simmental dairy cows and 39 from Sarda ewes) were collected within 6 h (T0) and after 24 h (T24) from parturition. Colostrum samples were subjected to the radial immunodiffusion (RID) assay for the quantification of IgG and to MilkoScan FT-plus for the estimation of protein content (TP, %), which was then used as an indirect method for the evaluation of colostrum quality. To compare the two methods, correlation and regression analysis of IgG quantification by RID and protein (%) content estimation by MilkoScan FT-plus data was performed using Procedure CORR and Procedure REG of SAS, respectively (version 9.3, SAS Institute Inc., Cary, NC, USA). Thresholds for the classification of good colostrum quality (as determined by RID assay, the gold standard method) were set at 50 g of IgG/L in cows and 20 g of IgG/L in ewes. The concentration of IgG in bovine colostrum assayed by RID showed a variation ranging from 41.45 to 199.97 g/L with an average of 99.85 ± 40.84 g/L at T0, and from 2.83 to 75.93 g/L with an average of 19.76 ± 19.01 g/L at T24. Regarding ovine colostrum, the concentration of IgG assayed by RID ranged from 34.45 to 156.32 g/L with an average value of 77.82 ± 37.58 g/L at T0, and from 5.6 to 69.74 g/L with an average of 27.90 ± 19.81 g/L at T24. Colostrum TP ranged from 3.70 to 23.96% for bovine colostrum and 6.32 to 22.88% for ovine colostrum using MilkoScan FT-plus. MilkoScan FT-plus and RID data were highly and significantly correlated (r = 0.91 for bovine and r = 0.94 for ovine colostrum), and regression analysis showed a strong relationship between IgG concentration provided by RID assay and TP provided by MilkoScan FT-plus (R^2^ = 0.84 and 0.88 for bovine and ovine, respectively). Optimal cut-off points for the greatest accuracy of TP (%) determined by MilkoScan FT-plus were 12.8% in cows [with 88.9% sensitivity (Se) and 100% specificity (Sp)] and 9% in ewes (with 96.7% Se and 100% Sp). In conclusion, these outcomes indicate that MilkoScan FT-plus as an indirect method may be a reliable tool for the estimation of the total IgG concentration and quality in bovine and ovine colostrum. Moreover, the cut-off levels of 12.8% for bovine and 9% for ovine of TP, seem sufficient to ensure that all poor-quality colostrum can be classified as such, with only a low proportion of good-quality colostrum being misclassified as poor-colostrum, thereby increasing the probability of delivering good-quality colostrum to new-born calves and lambs.

## 1. Introduction

Colostrum, the first mammary secretion after parturition, is an important source of nutrition and passive immunity, which ensures ruminant offspring protection in early life [1,2,3]. The placenta barrier does not allow the passage of Ig from the dam to the fetus; therefore, the offspring must depend entirely on antibodies received via colostrum [4,5].

Insufficient consumption and absorption of Ig result in the failure of transfer of passive immunity (FTP), defined as serum IgG < 10 mg/mL [5] and <15 mg/mL [6,7,8] in calves and lambs, respectively.

In calves and ewes, prevention of FTP is achieved by timely feeding of adequate quantities of colostrum that contains a minimum of 50 g of IgG/L [3,9] and 20 g of IgG/L, respectively [9].

Colostrum composition and its quality are affected by several factors, such as breed, parity, nutritional and health status, season, dry period length, time of milking postpartum, and individual farm [10,11].

The quality of colostrum correlates directly with the amount of IgG it contains [2]. The concentration of IgG in colostrum varies between and within breeds and, in ruminants, is often insufficient to provide passive immunity during the neonatal period [12].

The measurement of colostrum quality must be reliable, accurate and easy to perform, and dairy producers should measure colostral IgG before feeding it to newborns or storing it for later use. To date, several methods have been developed to determine, especially in bovines, colostral IgG concentration. These methods include ELISA [13,14]; electrophoresis [15]; and traditional methods, such as the use of a colostrometer [16,17] or Brix refractometer [18]. Radial immunodiffusion (RID), developed by Mancini et al. [19], is currently the gold standard for determining IgG concentration in maternal colostrum and serum [20]; however, the execution time using this method can range from 18 to 48 h depending on the test kit, so producers are unable to test colostrum quality prior to gut closure. Recently, infrared spectroscopy (IR) has emerged an alternative technique for assessing colostrum quality [14,21,22]. Near infrared spectroscopy (NIR) has been reported as a possible fast method for measuring IgG concentration in colostrum [14,21]. From this, the authors concluded that mid-infrared spectroscopy (mid-IR spectroscopy) has potential application in the assessment of Ig in colostrum, although the best alternative to predict IgG concentration in bovine colostrum in regard precision remains the refractometer. Elsohaby et al. [23] used IR in combination with partial least squares analysis (PLS) to assess colostral IgG concentration in dairy cows, underlining that IR is a rapid and accurate method for assessing colostrum quality.

MilkoScan FT-plus is based on Fourier transform infrared technology spectroscopy, and in the dairy industry, the MilkoScan FT-plus analyzer is routinely used to measure the chemical constituents of milk, providing a range of compositional parameters, including, for example, fat, protein, lactose, urea, BHB and fatty acids [24]. In particular, IgG represents the major class of colostral Ig in ruminants, reaching 94% and 80–90% of the total Ig content in the ovine and bovine colostrum, respectively [25,26]. Therefore, total protein (TP) assayed by MilkoScan FT-plus may provide an estimation of total Ig in colostrum, since Ig is included in the determination of total protein.

The development of a colostrum quality assessment method that can rapidly and accurately determine Ig concentration would be of great benefit to producers, as poor colostrum could be identified early within the first day of life, and the neonate could receive additional Ig or a further supplementation of good colostrum. While a few published works have evaluated colostrum with the MilkoScan FT-plus analyzer [14,27], none of them focused on immunoglobulin estimation.

The objectives of our study were to determine whether MilkoScan FT-plus could predict colostral Ig concentration and to confirm whether this method has good accuracy in the determination of good or poor colostrum quality and the IgG content of colostrum in bovine and ovine species.

## 2. Materials and Methods

### 2.1. Sample Collection

A total of 94 colostrum samples, 54 from Simmental dairy cows and 39 from Sarda ewes, were collected from two farms between April and May 2016. At each farm, each animal was milked in the milking parlor, and all the milk produced was collected in a bucket from which the owner or an employee harvested the colostrum samples into plastic tubes within 6 h (T0) and after 24 h (T24) from parturition. At the end of the collection period at the farms, bovine colostrum samples were collected from 27 cows (27 samples for the T0 time point and 27 samples for the T24 time point), whereas ovine colostrum samples were collected from 21 ewes (18 samples for the T0 time point and 21 samples for the T24 time point). We are aware that samples collected at T24 should not be defined as colostrum; however, we decided to also collect these samples in order to have samples with a low concentration of IgG and to increase our sample number since the number of subjects available was relatively low. From each harvesting period, 100 mL of colostrum was sampled, and tubes were then inverted 8 to 10 times to thoroughly mix the colostrum for accurate homogenization. Samples were stored at −20 °C until transportation to the Laboratory, Magna Græcia University of Catanzaro, where they were stored at −80 °C until analysis. Before the analysis, colostrum samples were thawed at room temperature (20–24 °C), and an aliquot (approximately 50 mL) was transferred into a plastic test tube with a smaller volume for measurements of IgG content by RID. The remaining aliquot was used for protein measurement by Foss MilkoScan FT-plus (Foss Electric A/S, Hillerød, Denmark).

### 2.2. Colostrum Analysis

Colostrum samples were thawed at room temperature (20–24 °C) and vortexed for 10 s to ensure good homogeneity. The IgG concentration was assessed via the radial immunodiffusion technique using the Ovine and Goat IgG IDRing^®^ Test (IDBiotech, Immuno Diffusion Biotechnologies SARL, Issoire, France) and Bovine IgG IDRing^®^ Test (IDBiotech, Immuno Diffusion Biotechnologies SARL, Issoire, France). Briefly, 50 µL of colostrum was diluted in 4950 µL of physiological solution (dilution 1), and then 50 µL of dilution 1 was diluted in 450 µL of SRID buffer 1X delivered by the manufacturer. The plate wells were filled with 15 µL of sample, reserving 4 wells to the corresponding standards delivered by the manufacturer. A total of 12 plates were used. Plates were placed in a humid box and incubated for 20 ± 2 h at 37 °C. Diffusion was stopped by adding 5 mL of freshly prepared 2% acid acetic solution and leaving the plates for 1 min at room temperature. Plates were rinsed twice with deionized water, and after adding 5 mL of deionized water, they were incubated for 15 min at room temperature. The resulting ring diameters surrounding the well were measured using the IDRing^®^ Viewer system (IDBiotech, ImmunoDiffusion Biotechnologies SARL, Issoire, France). A standard curve was established by plotting the square root concentration on the abscissa against the diameters for each standard on the ordinate (R2 of each plate was between 0.98 and 0.99). A standard curve was generated using a reference serum supplied by the manufacturer (200, 100, 50, 25 μg/mL), and IgG concentration was calculated using regression analysis. The standards were calibrated with an IgG1 solution purified from bovine and ovine colostrum, and the range and the upper and lower limits of detection were 200 and 25 μg/mL, respectively.

All colostrum samples were thawed at room temperature (20–24 °C) and vortexed for 10 s to ensure an adequate homogeneity. Colostrum samples were diluted with deionized water (1:1), warmed at 40 °C in a water bath and analyzed for protein content using MilkoScan FT-plus (Foss Electric, Hillerød, Denmark). Samples were analyzed in triplicate. Protein content (%) was measured using an improved milk calibration for each species (Foss Electric Application Note, 2002).

The calibrated curve for parameters was updated for intercept and slope using the milk calibration set obtained from the Milk Standard Laboratory of the Associazione Italiana Allevatori (Maccarese, Rome, Italy).

### 2.3. Statistical Analysis

To compare the two methods, correlation analysis of RID and protein (TP, %) was performed using MilkoScan FT-plus data and Procedure CORR of SAS (version 9.3, SAS Institute Inc., Cary, NC, USA). In addition, linear regression analysis of MilkoScan FT-plus and RID data was performed using Procedure REG of SAS. The TP (%) of each colostrum sample (n = 54 for bovine and n = 39 for ovine) estimated using MilkoScan FT-plus was plotted against the IgG concentration measured by RID. Statistical significance was declared at *p* < 0.05.

**Figure 1:**
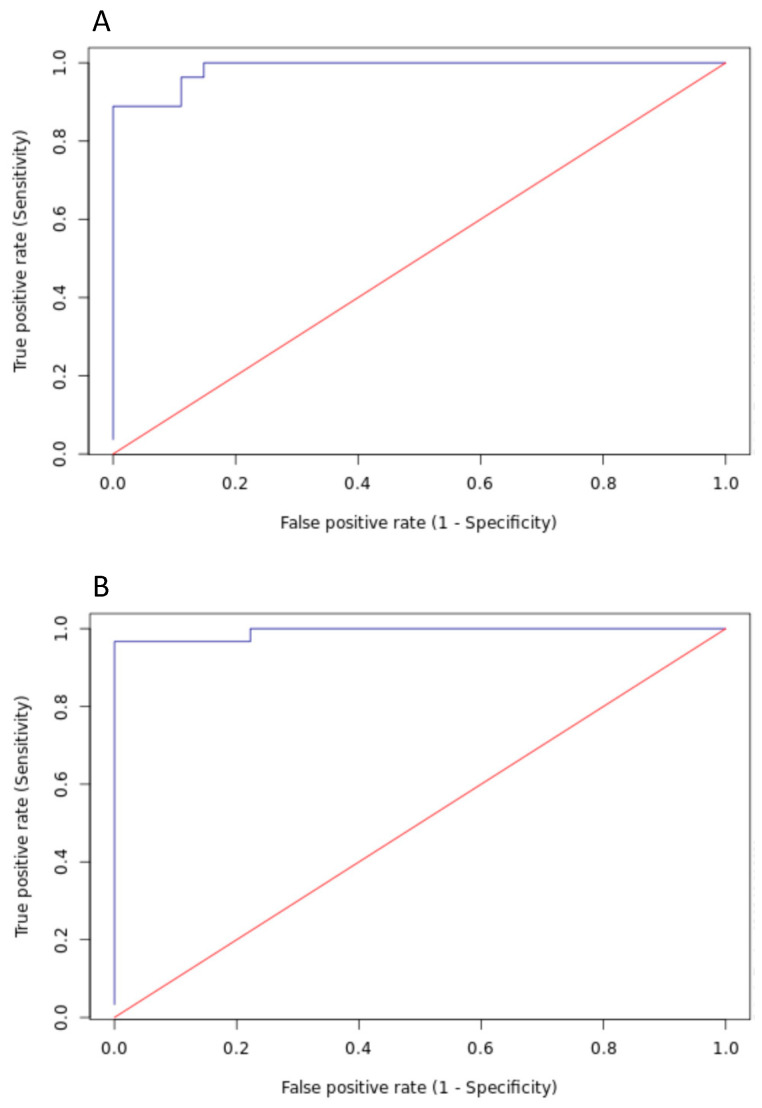
Receiver operating characteristic (ROC) curves of the diagnostic accuracy of MilkoScan FT-plus to detect colostrum of good quality for bovine ((**A**); IgG ≥ 50 g/L) and for ovine ((**B**); IgG ≥ 20 g/L). The values for the area under the curve (AUC) are 0.986 and 0.993, respectively, for bovine and ovine colostrum.

To examine the performance of the MilkoScan FT-plus method, receiver operating characteristic (ROC) curves to calculate diagnostic accuracy of the MilkoScan FT-plus method in determining colostrum of the 2 different species were created using the analysis tool Sigmaplot (Figure 1). In addition, various accuracy estimates at predefined MilkoScan FT-plus cut-off values of sensitivity (Se), specificity (Sp), respective 95% confidence intervals, positive (PPV) and negative predictive values (NPV) and Youden’s index were automatically calculated during the ROC curve analysis in Sigmaplot. The PPV was the proportion of test-positive samples that truly had IgG < 50 g/L for bovine and IgG < 20 g/L for ovine, and NPV was the proportion of test-negative samples that truly had IgG ≥ 50 g/L for bovine and IgG ≥ 20 g/L for ovine.

## 3. Results and Discussion

Descriptive statistics of IgG values obtained by RID, and the percentage of TP obtained by MilkoScan FT-plus are showed in Table 1. The concentration of IgG in bovine colostrum assayed by RID showed a variation ranging from 41.45 to 199.97 g/L with an average of 99.85 ± 40.84 g/L at T0, and from 2.83 to 75.93 g/L with an average of 19.76 ± 19.01 g/L at T24. Regarding ovine colostrum, the concentration of IgG assayed by RID ranged from 34.45 to 156.32 g/L with an average value of 77.82 ± 37.58 g/L at T0, and from 5.6 to 69.74 g/L with an average of 27.90 ± 19.81 g/L at T24.

**Figure 2:**
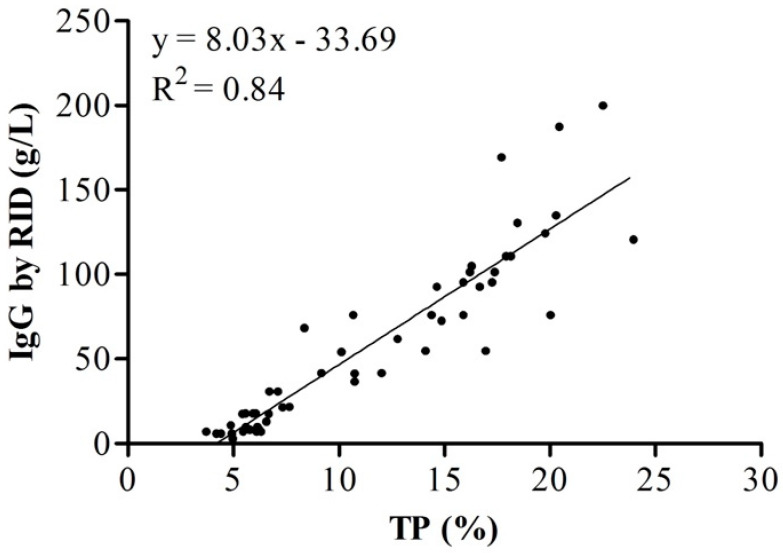
Relationship between total protein (TP) and immunoglobulin G (IgG) in bovine colostrum. IgG was measured by radial immunodiffusion, and total protein was measured by MilkoScan FT^+^.

**Figure 3:**
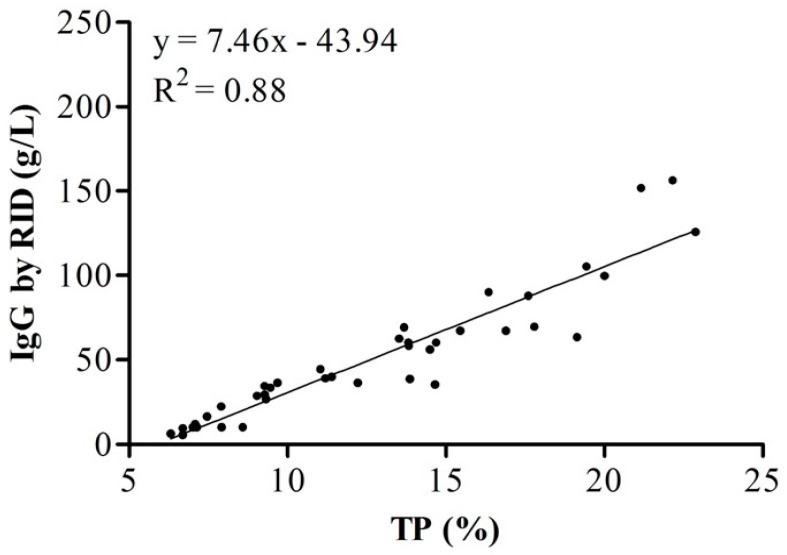
Relationship between total protein (TP) and immunoglobulin G (IgG) in ovine colostrum. IgG was measured by radial immunodiffusion, and total protein was measured by MilkoScan FT^+^.

The correlation coefficient between RID and MilkoScan FT-plus values was r = 0.94 for ovine and r = 0.91 for bovine colostrum, and the linear regression analysis showed a strong relationship between these two methods (R^2^ = 0.84 for bovine and R^2^ = 0.88 for ovine colostrum; *p* < 0.0001; Figure 2 and Figure 3). The correlation coefficient of 0.91 in bovine was slightly higher than 0.85 found by Løkke et al. [14], who used mid-IR spectroscopy in colostrum samples of Holstein cows. The mean IgG concentration of samples collected from cows upon first milking after parturition was similar to that reported by Bielmann et al. [28] (94.4 g/L) but greater than reported by Kehoe et al. [29] (34.9 ± 12.23 g/L) and by Chigerwe et al. [30] (68.5 ± 32.4 g/L) in colostrum of cow breeds different from the present study. The mean IgG concentration of samples collected from ovine upon first milking was similar to that in previous research in which it was assayed by RID [31,32], with values of 79 ± 5.6 and 82.1 g of IgG/L, respectively. Several factors affect IgG concentration in both species, such as breed, parity [11,33], time of sampling [34] volume of colostrum and method of feeding [8], and these may explain the variation in IgG levels across these studies. In addition, our results suggest that IgG decreases over time through the 24 h after parturition. As previously reported [20,34], colostrum collected for more than 3 h after parturition has lower IgG concentration than colostrum collected within a shorter period. It is well known that the highest IgG concentration of colostrum is at 1 h post-partum, as it rapidly declines at 12 h and 24 h post-partum [34]. Løkke et al. [14] included natural variation, such as fat and lactose, to predict IgG. The authors showed that the correlation between protein concentration and IgG was r = 0.70, underlining that FT-IR prediction was more dependent on protein than IgG, and the latter cannot be distinguished from the signals of other proteins in a complex mixture of constituents as colostrum. The authors concluded that although FT-IR is useful for the prediction of IgG, the prediction is far from perfect, and the refractometer is the best alternative among the tested methods. Riviero et al. [21] used NIR for the prediction of IgG concentration (R^2^ = 0.95 in calibration and 0.94 in cross-validation), but no comparison between TP and IgG was found. Elsohaby et al. [23] reported a correlation coefficient of r = 0.88; however, the calibration model used for prediction of IgG was previously built using a different set of colostrum samples used in a previous study [22].

Our results showed a higher correlation than that reported by Bartier et al. [17], who compared indirect methods, such as the use of the colostrometer or Brix refractometer, with IgG assayed by RID (r = 0.77). Other authors [35] showed that the specific gravity of colostrum, as measured by the colostrometer, had a good correlation with IgG concentration determined by RID (r = 0.70). The wide range of reported correlation coefficients could be explained by the variation in the source of colostrum and in the number of samples.Furthermore, we must consider the fact that, comparison between different studies it is often not suitable, because very various technologies and methods are used to identify IgG in colostrum.. Indeed, non-IgG protein could affect the variation in the colostrometer and Brix methods [20].

In a recent study by Kessler et al. [9], using an optical Brix refractometer, Brix values ranged from 11.4 to 34.6% and 15.4 to 40.0% in bovine and ovine colostrum, respectively.

In both species, Brix was highly correlated with IgG and protein concentrations (bovine, r = 0.98; ovine, r = 0.87) [9]. The Brix refractometer measures the total solutions dissolved and not specifically Ig, so differences in colostrum nutrient contribuite to a variation in the final estimate. On the other hand, commercial kits for the estimation of ovine and bovine IgG based on ELISA, RID or other assays are usually supplied with their own proprietary reference materials that are commonly derived from serum and designed for IgA, IgG and IgM. However, these methods may lead to erroneous quantification of IgG in immune methods because different subclasses of IgG derive from serum [36].

Considering this, MilkoScan FT-plus indirectly evaluates the total percentage of proteins in colostrum, and, therefore, it could be used to ensure good prediction of IgG. The aim of an optimal test for Ig quantification is to guarantee that only high-quality colostrum is used to feed new-born ruminants. To ensure this, it is important to use appropriate cut-off levels that correspond as much as possible to the real Ig values. Appropriate cut-off points need to be determined to ensure that only good-quality colostrum is fed to newborns and only poor colostrum discarded. Thresholds for discriminating good and poor colostrum quality refer to IgG concentrations analyzed by RID as reference. With respect to established threshold values in scientific reports, we used cut-off points of 50 g/L for colostrum in cows [11,28,37] and 20 g/L in ovine [4,9,38,39].

For the determination of species-specific appropriate TP by MilkoScan FT-plus, cut-off points to differentiate good from poor colostrum quality, Sp, Se, PPV, NPV, and further accuracy measures were calculated with reference to the RID assay thresholds (Table 2). Based on the ROC curve analysis, maximizing Se and Sp outcomes, which in turn leads to maximizing the Youden index (Figure 1), the optimal cut-off points for TP by MilkoScan FT-plus were 12.8% in cows (with 88.9% Se and 100% Sp; ROC area under the curve: 0.986) and 9% in ovine (with 96.7% Se and 100% Sp; ROC area under the curve: 0.993). Similar to the ROC curve analysis, the Youden index, indicating the greatest sum of Se and Sp calculated at predefined MilkoScan FT-plus cut-offs, was highest at 12.8% in cows and 9% in ewes. The Youden index measures the effectiveness of a diagnostic marker and enables the selection of an optimal threshold value (cut-off points) for the respective marker. Maximizing Se allows for the identification of the highest proportion of adequate colostrum that is truly good quality. However, a concomitantly low Sp leads to a misclassification of truly inadequate colostrum, which is not detected as colostrum when the IgG concentration is below 20 g/L.

The sensitivity of a test is the probability to detect colostrum samples with low quality, whereas specificity is the probability that a good-quality (≥50 g/L for cows and ≥20 g/L for ewes) sample is correctly identified. The highest combined sensitivity and specificity occurred at 12.8% for bovine and 9% for ovine (Table 2).

Positive (PPV) and negative predictive values (NPV) are strictly linked to the type of sample and indicate the probability that a test-positive or test-negative sample is truly of poor or adequate quality, respectively, given the prevalence in the sample population. That means that at both suggested cut-offs (12.8% for bovine and 9% for ovine), the potential of MilkoScan FT-plus to detect poor-quality colostrum in both bovine and ovine was 100% (all poor-quality colostrum samples were detected), leading to the true poor-quality colostrum being discarded. On the other hand, in bovine colostrum, the chance of identifying a good-quality colostrum was 90.01%, leading to the 10% probability of good-quality colostrum being discarded, whereas in ovine colostrum, the chance of identifying good-quality colostrum was 98.61%, resulting in a promising and efficient method, since the chance of discarding good-quality colostrum was very low. Based on this, TP estimation using MilkoScan FT-plus showed high performance in the diagnostic test, resulting in better performance than that of the Brix refractometer and colostrometer, both on-farm tools [9,17,40]. In particular, these authors argue that a substantial number of good quality colostrum would be misclassified as poor-quality colostrum and that this misclassification would be more pronounced in farms with a low percentage of poor-quality colostrum.

## 4. Conclusions

Although between-species variations in colostrum composition were identified in the present study, determination of TP using MilkoScan FT-plus can be considered an acceptable method for the estimation of colostral IgG concentration in bovine and ovine species. TP determination values using MilkoScan FT-plus had a strong relationship with IgG in the colostrum of all species. Our observations confirm the suitability of common IgG thresholds and MilkoScan FT-plus cut-off values for the classification of colostrum quality. The rapid evaluation of colostrum quality and IgG concentration can be used to improve farm management and animal health. Thus, MilkoScan FT-plus can, in turn, be used as an indirect and rapid method, as well as being an accurate, precise and economical method for estimating the IgG concentration and quality in bovine and ovine colostrum.

The present study is the first step toward improving the assessment of colostrum quality at the farm level.

## Figures and Tables

**Table 1:** Descriptive statistics for colostrum samples analyzed by radial immunodiffusion (RID) and MilkoScan FT^+^.

Item	Time	Mean	SD	95% CI	Min	Max
Bovine Colostrum						
TP ^1^ (%)	0 h	16.67 ^a^	3.33	15.38–17.96	9.18	23.96
	24 h	6.42 ^b^	1.81	5.71–7.14	3.7	10.74
IgG ^2^ (g/L)	0 h	99.85 ^a^	40.84	84.31–115.14	41.45	199.97
	24 h	19.76 ^b^	19.01	12.24–27.28	2.83	75.93
Ovine Colostrum						
TP ^1^ (%)	0 h	16.05 ^a^	4.12	14.00–18.01	9.27	22.88
	24 h	9.87 ^b^	3.35	8.35–11.39	6.32	10.79
IgG ^2^ (g/L)	0 h	77.82 ^a^	37.58	59.14–96.52	34.45	156.32
	24 h	27.90 ^b^	19.81	18.88–36.92	5.6	69.74

^a,b^ Means with different superscripts differ between 0 and 24 h (*p* ≤ 0.05). ^1^ Total protein. ^2^ Immunoglobulin G.

**Table 2:** Sensitivity (Se), specificity (Sp), Youden index, positive predictive value (PPV), and negative predictive value (NPV) for different MilkoScan FT-plus cut-off values compared with values of 50 g/L of IgG in bovine colostrum (n = 54) and 20 g/L of IgG in ovine colostrum (n = 39), measured by RID assay (gold standard test).

Species	Cut-Point Value, % Proteins by FT-IR	Se, % (95% CI)	Sp, % (95% CI)	Youden Index ^1^	PPV % ^2^	NPV % ^3^
Bovine	8.40%	100 (87.50–100)	85.20 (67.50–94.1)	0.852	87.11	100
	10.10%	96.30 (81.70–99.30)	88.90 (71.90–96.10)	0.852	89.66	96.00
	10.70%	92.60 (76.60–97.90)	88.90 (71.90–96.10)	0.815	89.30	92.30
	12.00%	88.90 (71.90–96.10)	96.30 (81.70–99.30)	0.852	96.00	89.66
	12.80%	88.90 (71.90–96.10)	100 (87.50–100)	0.889	100	90.01
	14.40%	81.50 (63.30–91.80)	100 (87.50–100)	0.815	100	84.39
Ovine	7.90%	100 (88.60–100)	77.80 (45.30–93.70)	0.778	65.88	100
	8.60%	96.70 (83.30–99.40)	88.90 (56.50–98.00)	0.856	98.43	78.87
	9.00%	96.70 (83.30–99.40)	100 (70.10–100)	0.967	100	98.61
	9.30%	90.00 (74.40–96.50)	100 (70.10–100)	0.90	100	95.89
	9.70%	80.00 (62.70–90.50)	100 (70.10–100)	0.80	100	92.11

Positive value is detection of <50 g of IgG/L in bovine colostrum samples and <20 g of IgG/L in ovine colostrum samples. ^1^ Youden index = [(Se + Sp)/100] − 1. ^2^ PPV = positive predictive value. ^3^ NPV = negative predictive value.

## Data Availability

Data available on request from the authors.
